# Three-Dimensionally Structured Flexible Fog Harvesting Surfaces Inspired by Namib Desert Beetles

**DOI:** 10.3390/mi10030201

**Published:** 2019-03-22

**Authors:** Jun Kyu Park, Seok Kim

**Affiliations:** Department of Mechanical Science and Engineering, University of Illinois at Urbana-Champaign, 1206 W. Green, St. Urbana, IL 61801, USA; jpark323@illinois.edu

**Keywords:** Namib desert beetles, biomimetics, fog harvesting, wettability gradient, shape gradient

## Abstract

Fog harvesting of the Namib desert beetles has inspired many researchers to design artificial fog harvesting hybrid surfaces, which commonly involve flat hydrophilic patterns on hydrophobic surfaces. However, relatively less interest has been shown in the bumpy topography of the Namib desert beetle’s dorsal surface as well as its curved body shape when designing artificial hybrid surfaces. In this work, we explore a fog harvesting flexible hybrid surface that has a superhydrophilic 3D copper oxide pattern on a hydrophobic rough elastomer background surface enabled by transferring a copper layer from a prepared donor substrate to a receiving elastomer substrate. The water collection rates of the hybrid surface and control samples are measured, and the results reveal the advantages of 3D bumpy structures on a curved shape surface to facilitate fog harvesting, particularly in more unfavorable fog stream conditions. The curved 3D bumpy hybrid surface exhibits an over 16 times higher water collection rate than the flat 2D hybrid surface in the fog stream in parallel to the hybrid surface. This work provides an improved understanding of the role of the Namib desert beetle’s bumpy dorsal surface and curved body shape, and offers an insight into the design of novel surfaces with enhanced fog harvesting performance.

## 1. Introduction

**Figure 1:**
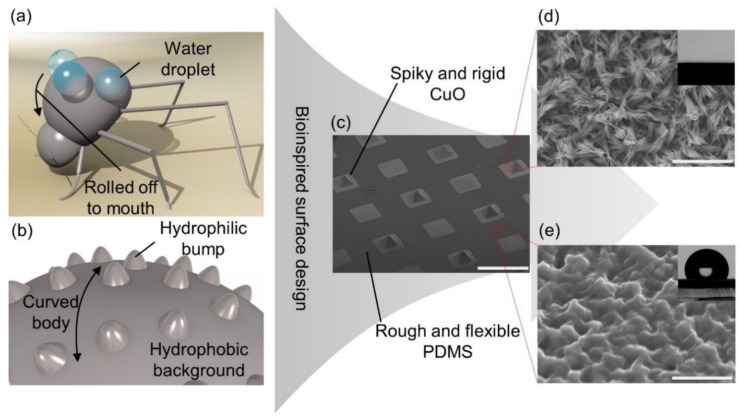
A schematic overview of the bioinspired hybrid surface. (**a**) A cartoon of a fog harvesting beetle. (**b**) A cartoon of the magnified dorsal surface of a beetle. (**c**) A scanning electron microscope (SEM) image of the fabricated flexible hybrid surface. The scale bar indicates 1 mm. (**d**) A magnified SEM image of a CuO region. The inset is a spread water droplet on it. The scale bar indicates 5 μm. (**e**) A magnified SEM image of a rough PDMS surface. The inset is a water droplet on it. The scale bar indicates 5 μm.

Water is the most abundant resource in nature. However, the portable water available for living organisms is rather confined, and the amount is constantly diminishing. On top of that, accessible fresh water is unevenly distributed over the surface of the earth. Water scarcity is more severe in arid areas, and living creatures in such areas must adopt special abilities to collect water from atmospheric air for survival [1,2,3]. For instance, located in the southern Africa coast, the Namib Desert is one of the driest areas on the earth and the only sustainable water resource is fog from the Atlantic Ocean. Thus, some living creatures in the Namib Desert adopt several strategies to collect water from the foggy air. In particular, several species among the Namib desert beetles have been reported to tilt their bodies toward the foggy wind to collect water droplets on their dorsal surfaces [4,5,6]. In such a way, they can harvest water mist effectively due to the presence of a wax-coated, hydrophobic dorsal surface, which has a few hundred microns tall hydrophilic bumps without wax (Figure 1a,b). The fog colliding with or condensing on the wax-coated hydrophobic surface (contact angle (CA) > 90°) is propelled to the hydrophilic bumps, and droplets settling on the hydrophilic bumps (CA < 90°) continuously grow. The grown water droplet eventually covers the entire hydrophilic bump and becomes large enough to be ready to roll down to the mouth. Inspired by the Namib desert beetles, Parker et al. successfully demonstrated the fog harvesting capabilities of their artificial hybrid surfaces consisting of hydrophilic patterns on hydrophobic backgrounds [6]. After that, many researchers have developed their artificial fog harvesters with various fabrication techniques by focusing on fabricating flat hybrid surfaces with high wettability difference between superhydrophilic (CA < 5°) patterns and superhydrophobic (CA > 150°) backgrounds [7,8,9,10,11,12]. 

While many efforts have been made on designing artificial hybrid surfaces with a large spatial wettability difference, there has been relatively little work done on 3D structured and curved fog harvesting surfaces mimicking the actual hydrophilic bumps and the curved body of Namib desert beetles. For example, there have been only a few previously reported flat hybrid surfaces with 3D bumpy structures although those are not morphologically comparable to the actual bumps of Namib desert beetles [13,14]. Therefore, it is worthwhile to study the contribution of the bumps and the curved dorsal surface of Namib desert beetles to fog harvesting using artificial curved hybrid surfaces with 3D bumpy structures. Indeed, several reports have proven that the geometric discontinuity from 3D structures can significantly increase the vapor diffusion flux to facilitate condensation [15,16,17].

**Figure 2:**
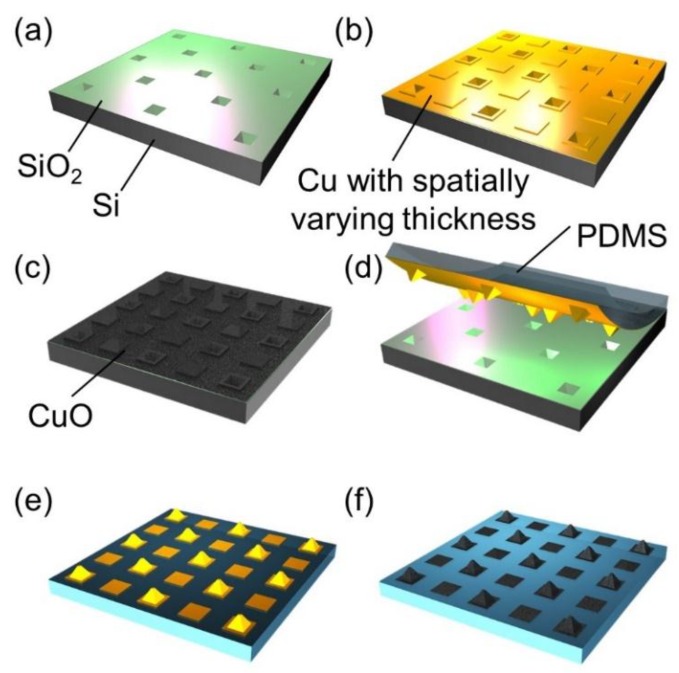
A schematic illustration of the fabrication process of the hybrid surface with a 3D superhydrophilic pattern. (**a**) SiO_2_ is thermally grown on a prepared Si donor substrate where a pyramidal pit array is formerly made. (**b**) A Cu layer with spatially varying thickness is deposited on the Si donor substrate by two step electroplating. (**c**) Cu is oxidized by alkaline oxidation and a CuO-Cu composite layer is formed on the donor substrate. (**d**) A PDMS precursor is poured and cured on the donor substrate to mold a PDMS receiving substrate. Then the CuO-Cu composite layer is peeled off from the donor substrate and transferred onto the PDMS receiving substrate. (**e**) The Cu pattern is created after timed etching of the CuO-Cu layer on the receiving substrate. (**f**) The additional oxidation converts the Cu pattern to the CuO pattern.

In this work, we explore a flexible hybrid surface with a 3D superhydrophilic copper oxide (CuO) pattern on a hydrophobic rough polydimethylsiloxane (PDMS) background, which is inspired by the bumps on the curved dorsal surface of Namib desert beetles (Figure 1c–e). To enable these morphological features of the flexible hybrid surface, a copper (Cu) layer prepared on a silicon (Si) or glass donor substrate is transferred to a PDMS receiving substrate, which is molded on and peeled from the donor substrate [18,19,20,21]. After the transfer, the Cu layer is patterned and oxidized to form a superhydrophilic CuO pattern on a hydrophobic rough PDMS substrate. As a result, the transferred and oxidized Cu layer on a PDMS substrate can have 2D or 3D patterns depending on the initial morphology of the donor substrate. For the successful transfer, the adhesion of a transferred material to a receiving substrate should be greater than that to a donor substrate. Therefore, most previously reported similar processes transferred several nanometer thin layers of noble metals, such as Au or Ag, that have weak adhesion to other materials and mechanical flexibility [18,19,20,21]. Thick non-noble metals, such as tens of micron thick Cu layers, used in this work are hard to peel off and transfer from a donor substrate due to the high adhesion of them to common donor substrate materials, including Si and glass, and their mechanical rigidity. To overcome this challenge, we developed a method to dramatically promote adhesion between a receiving PDMS substrate and a Cu layer by generating nanostructured CuO on the Cu layer, and thus increasing contact area in between significantly (Figure 2c–d). This approach allows the successful fabrication of flexible PDMS surfaces with various 2D or 3D CuO patterns, which are fog harvesting hybrid surfaces in this work. The fog harvesting performances of the fabricated hybrid surfaces with diverse patterns and morphologies are systematically characterized with a custom setup. Using experimental and numerical results, the effect of 3D superhydrophilic bumps on fog harvesting compared to 2D patterns as well as the advantage of a curved surface in the unfavorable fog stream direction are studied.

## 2. Materials and Methods

### 2.1. Materials

PDMS (Sylgard 184), Cu electroplating kits, including Microfill EVF Carrier, Brightner, and Leveler, were purchased from Dow Corning, Midland, MI, USA. Potassium hydroxide pellet (KOH) was purchased from Fisher Scientific, Waltham, MA, USA. AZ 5214 photoresist was purchased from MicroChemicals, Ulm, Germany. Trichloro(1H,1H,2H,2H-perfluorooctly)silane, NaClO_2_, NaOH, and Na_3_PO_4_·12H_2_O were purchased from Sigma Aldrich, St. Louis, MI, USA. Cu etchant (CE-100) was purchased from Transene, Danvers, MA, USA. Hydrochloric acid (HCl) was purchased from Macron Fine Chemicals, Allentown, PA, USA.

### 2.2. Experimental Details on Characterization

The scanning electron microscope (SEM) images of the hybrid surfaces were taken using an SEM (Hitachi S-4800, Schaumburg, IL, USA). The CA of water droplets (7 μL) on spiky CuO and rough PDMS surfaces were measured using a goniometer (CAM-200, KSV instrument, Gothenburg Sweden). The sequential images of fog harvesting surfaces were taken by a digital camera (EOS 100D with 100 mm macro lens, Canon, Tokyo, Japan) at the sample tilting angle of 90°.

### 2.3. Experimental Details on Water Collection Rate (WCR) Measurement

**Figure 3:**
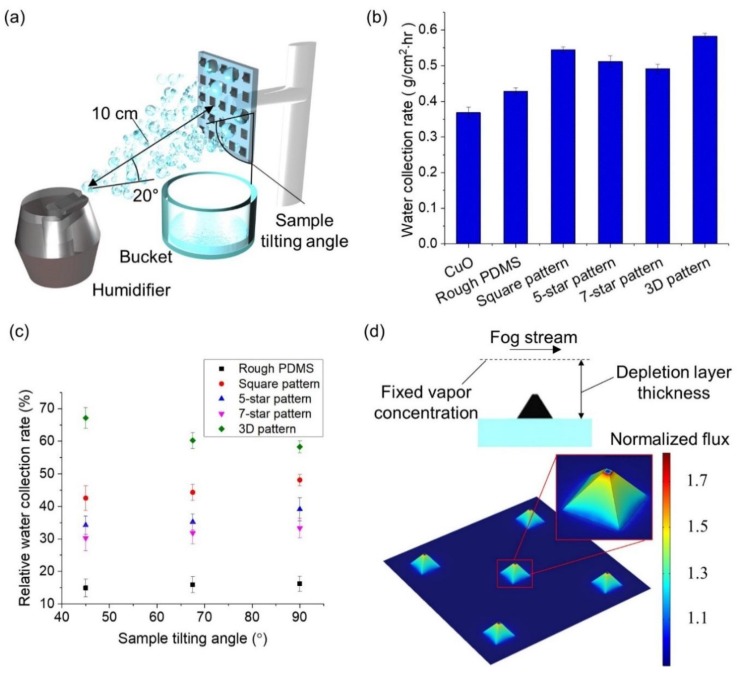
A schematic illustration of the experiment setup to measure the WCR and plots of the experimental and numerical results. (**a**) A schematic illustration of the setup used to measure the WCR of different samples. (**b**) The WCRs of different samples with a 90° sample tilting angle, including CuO, rough PDMS control samples and 2D square, 5-star, 7-star pattern samples and 3D pattern sample. (**c**) The relative WCRs of different samples with three different sample tilting angles of 45°, 67.5°, and 90°. (d) The FEA plot of the diffusion flux over the 3D pattern sample assuming a fog stream in parallel to the surface. The inset images show the conditions of FEA and the magnified plot over a 3D bump.

A commercially available ultrasonic humidifier (HUL520B, Honeywell, Charlotte, NC, USA) was used to examine the WCRs of those samples. The temperature and relative humidity of the surrounding were set to be 20 °C and 58%, respectively. The humidifier generating a fog stream with a 20° departure angle was placed 10 cm away from the sample stage and the collected water droplets were gathered in a bucket placed under the sample stage (Figure 3a). The samples were fixed on the stage with sample tilting angles of 45°, 67.5°, and 90° to the horizontal plane as represented in Figure 3a to mimic the fog harvesting stance of Namib desert beetles. The WCR was defined as a mass of collected water per hour divided by the sample surface area.

### 2.4. Finite Element Analysis (FEA) Details

In the FEA model, the fog stream was assumed to be in parallel to the 3D pattern sample surface (Figure 3d). The diffusion flux over the pyramidal bump and the surrounding flat region was numerically calculated. The depletion layer thickness was set as 10 mm based on previous studies and the vapor concentration at the top of depletion layer was maintained constant, which represents a mass source [15,16,22]. The sample surface was assumed as a mass sink and the diffusion flux was normalized by that on the flat region in the contour plot.

## 3. Results and Discussion

### 3.1. Fabrication of the Hybrid Surface with 3D and 2D Patterns

The fabrication of the hybrid surface with a 3D superhydrophilic pattern starts with the preparation of a reusable Si donor substrate with a pyramidal pit array. A 210 µm deep pyramidal pit array is formed by KOH etching on a Si substrate with a silicon nitride (Si_3_N_4_) etch mask. Then, the Si_3_N_4_ layer is removed and a silicon oxide (SiO_2_) layer is grown (Figure 2a). Additional details on the Si donor substrate preparation are found in Figure S1 in the supporting information. Here, the prepared Si donor substrate illustrated in Figure 2a can be used repeatedly as a template after the transfer process shown in Figure 2d, resulting in the fabrication load and cost reduction. Once a SiO_2_ layer is grown, a Cu layer with a selectively varying thickness is deposited on the Si donor substrate using two step electroplating with a photolithographically patterned photoresist (PR) layer (Figure 2b). Additional details on the electroplating procedure are found in Figure S2 in the supporting information. After this step, the Cu layer has two different thickness regions, i.e., a square pattern region with 95 μm thick Cu and a surrounding region with 25 μm thick Cu. Then, the top surface of the Cu layer is oxidized in a 90 ºC alkaline solution of NaClO_2_, NaOH, Na_3_PO_4_·12H_2_O, and DI water with a 3.75:5:10:100 weight mixing ratio, resulting in a CuO-Cu composite layer on the donor substrate (Figure 2c). Over the CuO-Cu composite layer, a PDMS precursor with a 10:1 pre-polymer to curing agent mixing ratio is poured to mold a PDMS receiving substrate. After the precursor is fully degassed and cured in an oven at 70 °C for 3 hours, the PDMS receiving substrate is carefully peeled off from the donor substrate and the CuO-Cu composite layer is completely transferred onto the PDMS receiving substrate (Figure 2d). It is worthwhile to note that the CuO after the oxidation has spiky nanostructures as shown in Figure 1d, which dramatically increases the contact area and consequently the adhesion between CuO and PDMS surfaces to overcome the adhesion between Cu and SiO_2_ surfaces during peeling. Indeed, the adhesion between CuO and PDMS surfaces proved to be highly strong based on our observation that the adhesion interface remains intact even when a PDMS substrate ruptures during unsuccessful peeling. After timed etching of the CuO-Cu composite layer that has a selectively varying thickness with Cu etchant and diluted HCl (5:1 volume ratio), the 3D Cu pattern is achieved on a PDMS surface(Figure 2e). The PDMS surface has a roughness that is created during the molding of the PDMS on the nanostructured CuO surface (Figure 2c,d) as shown in Figure 1e. An additional alkaline oxidation process to form spiky CuO nanostructures finalizes the fabrication process of the 3D superhydrophilic CuO pattern on the hydrophobic rough PDMS surface (Figure 2f). The fabrication of the hybrid surface with a 2D superhydrophilic pattern is precisely the same as that for the 3D pattern except that the donor substrate is a flat and smooth glass substrate. Hence, a Cu seed layer is directly deposited on the glass donor substrate and the 2D pattern is defined at the subsequent PR layer patterning and Cu electroplating step. Additional details on the fabrication procedure are found in Figure S3 in the supporting information. Figure 1c shows the SEM image of the fabricated hybrid surface composed of superhydrophillic CuO bumps and a hydrophobic rough PDMS background surface. The CuO bumps have highly spiky nanostructures as shown in Figure 1d. These nanostructures make the CuO surface superhydrophilic with CA below 5° even without any chemical treatment. The rough PDMS surface exhibits superior hydrophobicity with CA of 135° compared to a flat PDMS surface due to its surface roughness created during the molding process. The inset images in Figure 1d,e show water droplets on spiky CuO and rough PDMS surfaces.

### 3.2. Water Collection Process and WCR Measurement

To quantitatively characterize the fog harvesting abilities of the hybrid surface samples as a function of the superhydrophillic CuO pattern shapes, the water collection rate (WCR) is defined as the mass of collected water per hour divided by the sample surface area. Not only three samples with 2D square, 5-point star, and 7-point star CuO patterns, but also two control samples, including CuO and rough PDMS surfaces, are fabricated. Figure 3a illustrates the custom experimental setup to measure the WCRs of the samples. Sequential optical images of each hybrid surface sample during its water collection process are also available in Figure S4 in the supporting information. All samples are 2 cm by 2 cm large with 1.25 mm center-to-center spacing between neighboring pattern shapes in a square arrangement. As shown in Figure 3b, the CuO sample that is uniformly superhydrophilic over its entire area collects the least amount of water among all samples. The captured water droplets become a water film and adhere to the surface, which prevents the next water droplets from being efficiently captured and deteriorates condensation because of no dry area on the surface. A dry area would allow the nucleation of new droplets and expedite fog harvesting through condensation. In addition, a water film over the surface significantly reduces heat transfer across the surface, and thus, decreases fog harvesting efficiency [23,24]. The rough PDMS sample that is uniformly hydrophobic collects more water than the CuO sample because captured droplets can easily roll off the surface. However, there is no systematic water droplet coalescence that further facilitates water collection. Compared to these two control samples with spatially uniform wettability, the hybrid surfaces show higher WCRs because their heterogeneous wettability efficiently promotes water droplet coalescence. As soon as the humidifier is activated, tiny water droplets are captured on either the CuO regions or the PDMS background. Then, water droplets settling on the hydrophobic PDMS background of the hybrid surfaces tend to coalesce into bigger droplets and finally move to the superhydrophillic CuO region due to the wettability gradient. See the second and third columns of Figure S4 for the coalescence of droplets. In such a way, dry PDMS areas are maintained and the next water droplets continuously settle. The driving force to move water droplets toward the CuO region from the PDMS background that is caused by the wettability gradient is estimated as below [7,25,26,27]:

(1) $$F_{D}\approx \gamma_{w}(\cos\theta_{CuO}-\cos\theta_{PDMS})$$

Once a coalesced yet pinned droplet on the CuO region becomes big enough by further growth and coalescence with droplets on adjacent CuO regions, the droplet starts to fall or departs from the hybrid surface. It is also observed that the falling droplet captures other droplets on its moving path and releases additional fresh CuO pattern regions and PDMS areas for the next droplet settling. See the fourth and fifth columns of Figure S4 for falling droplets after further growth. Due to these combined effects, the hybrid surface samples show larger WCRs than the two other control samples. 

There is a secondary driving force to move surrounding water droplets into the inner area of the CuO region, which is caused by the shape gradient when the CuO pattern shape has sharp corners. A droplet on the sharp corner of the CuO region experiences asymmetric wetting because the contact area between a water droplet and the hybrid surface includes a larger rough PDMS portion when a water droplet covers the outer area. Thus, the CA of a water droplet on the inner area of the CuO region is lower than the CA on the outer area, which causes the asymmetric wetting. The secondary driving force by this shape gradient arising from the sharp corner is also estimated as below [7]:

(2) $$F_{D2}\approx \gamma_{w}(\cos\theta_{inner}-\cos\theta_{outer})$$

Indeed, square, 5-pointed star, and 7-pointed star pattern samples are designed and fabricated to utilize this shape gradient and optimize the WCR while each pattern shape maintains a constant area. Figure 3b shows that the square pattern sample has a superior WCR over the 5-pointed star as well as the 7-pointed star pattern samples and the WCR decreases as the corner becomes sharper. This can be explained by the relatively reduced *F_D_*_2_ associated with the 5-pointed and 7-pointed star pattern samples since a sharper corner results in smaller difference between *θ_inner_* and *θ_outer_* assuming that the droplet size is comparable to the pattern corner size. 

In comparison to the 2D samples, which represent conventional fog harvesting surfaces with planar patterns, the hybrid surface with a 3D pattern is fabricated using the process depicted in Figure 2. The 3D pattern sample has 210 µm tall pyramidal bumps and planar 2D squares in a staggered manner as shown in Figure 1c. The WCR of the 3D pattern sample is characterized in the same manner as that for the 2D samples since the pyramidal bumps do not increase the overall surface area meaningfully (less than 5% increase). Figure 3b indicates that the 3D pattern sample shows an even greater WCR than any other 2D samples. In agreement with the positive relationship between the WCR and the departure time, Figure S4 reveals that the droplet departure time is the shortest on the 3D pattern sample and then on the 2D square, 5-point star, and 7-point star pattern samples sequentially. It is also worthwhile to note that the diameter of a water droplet on the 3D pattern sample is smaller than that on other samples at the departure time, which implies that a droplet grows more out of plane on the 3D pattern sample (Figure S4d). This moves the center of mass of a pinned droplet further away from the surface, which may facilitate water droplet departure as well. These results evidently highlight the advantage of 3D pattern structures over 2D pattern shapes for water collection.

The effect of 3D pattern becomes more obvious when the WCR of each sample is measured with lower sample tilting angles as exhibited in the relative WCR plot in Figure 3c. In this plot, the relative WCR is defined as the percent increase in the WCR compared to the WCR of the uniformly superhydrophilic CuO sample at each sample tilting angle as summarized in Equation 3. The relative WCRs in Figure 3c are measured with sample tilting angles of 45°, 67.5°, and 90°:

(3) $$\mathrm{relative}\;WCR_{Surface}=\frac{WCR_{Surface}-WCR_{CuO}}{WCR_{CuO}}\times 100\;(\%)$$

As opposed to other samples, the relative WCR of the 3D pattern sample increases as the sample tilting angle decreases, which evidently shows that the favorable characteristics of bumpy structures become more obvious as the fog harvesting condition becomes more unfavorable with a lower projection area facing the fog stream and a weaker gravitational force. The enhanced WCR of the 3D pattern sample is mainly associated with the increased diffusion flux at the geometric discontinuity, i.e., bumpy structures. To verify this, FEA is utilized to visualize the diffusion flux over a pyramidal bump on the 3D pattern sample. As shown in Figure 3d, the diffusion flux over the pyramidal bump is much higher than that over the flat region. The enhanced diffusion flux as well as the direct collision of tiny water droplets in the fog stream with the 3D bumps result in the superior WCR of the 3D pattern sample over other 2D pattern samples. 

### 3.3. WCR on a Curved Hybrid Surface

**Figure 4:**
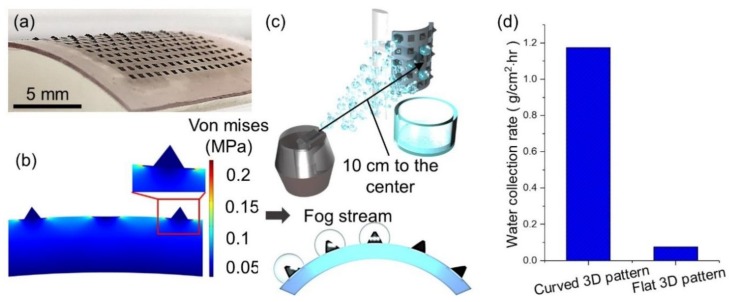
A curved 3D pattern sample and its fog harvesting performance. (**a**) An optical image of the curved 3D pattern surface. (**b**) A stress analysis plot of the curved 3D pattern surface. (**c**) A schematic illustration of experiment setup to measure the WCR with a fog stream in parallel to the sample. (**d**) The WCRs of the 3D pattern sample when it is curved and flat.

The other unique feature of the hybrid surface in this work is its mechanical flexibility, such that the hybrid surface can be wrapped over a curved object as shown in Figure 4a. The stress analysis shows that the von Mises stress is peaked not in the CuO pattern regions, but on the PDMS background when the hybrid surface is curved. This beneficial stress condition assists damage free and easy curving of the hybrid surface (Figure 4b). The curvature of the hybrid surface further promotes the WCR even in unfavorable situations with a lower projection area facing the fog stream. Indeed, the fog stream in the Namib Desert normally does not blow always in the normal direction to the dorsal surface of Namib desert beetles. Assuming the extreme situation, i.e., the fog stream in parallel to the fog harvesting surface, the curved surface nevertheless has a certain projection area facing the fog stream while a flat counterpart does not. This simple yet significant difference between curved and flat surfaces makes the Namib desert beetle’s water collection less sensitive to the fog stream direction. To validate this hypothesis, the WCR of a curved 3D pattern sample is measured while the humidifier is located 10 cm away from the sample center and in parallel to the sample stage as Figure 4c illustrates. The measured WCR of the curved 3D pattern sample is about 16 times larger than that of the flat 3D patterned sample as listed in Figure 4d. In addition, the WCRs of the curved and flat 3D pattern samples are also measured when the fog stream is perpendicular to the sample surface and it was found that there is no discernable difference of the WCR between curved and flat 3D pattern samples. These results undoubtedly represent that the hybrid surface, when curved, can significantly increase the WCR in unfavorable fog stream conditions without compromising its WCR in favorable fog stream conditions. This may be the reason why a fog harvesting creature takes advantage of having a round body and why bioinspired fog harvesters need to adopt these morphological advantages, accordingly.

## 4. Conclusions

In summary, Namib desert beetle-inspired hybrid surfaces composed of superhydrophilic CuO 2D or 3D patterns on flexible hydrophobic rough PDMS background surfaces were designed, fabricated, and characterized. The systematic experiments validate that 3D pattern hybrid surfaces exhibit superior water collection performance to 2D pattern hybrid surfaces. In addition, the flexible nature of the hybrid surface enables the study of the contribution of the curved surface shape to the WCR in unfavorable fog stream directions. The fabrication processes in this work involved no use of toxic chemicals, such as silane or fluoropolymers, coated on the water collecting surfaces for practical safer fog harvesting. This work provides an improved understanding of the role of bumpy structures and the rounded shape of the Namib desert beetle’s dorsal surface for fog harvesting, and thus, offers a guide to design 3D flexible fog harvesting surfaces.

## Supplementary Materials

The following are available online at https://www.mdpi.com/2072-666X/10/3/201/s1, Figure S1: The fabrication procedure to prepare a Si donor substrate with a pyramidal pit array, Figure S2: The fabrication procedure to produce a Cu layer with spatially varying thickness, Figure S3: The fabrication procedure to prepare the hybrid surface with a 2D superhydrophilic pattern, Figure S4: In situ observation of the water collection process of the hybrid surfaces.
