# Supplementary material for: Three-Dimensionally Structured Flexible Fog Harvesting Surfaces Inspired by Namib Desert Beetles
Source: Micromachines (Basel). 2019 Mar 22;10(3):201. doi: 10.3390/mi10030201 (PMC6470850; doi:10.3390/mi10030201)
Supplement: Supplementary file 1 [file micromachines-10-00201-s001.pdf]

Supporting Information: Three-Dimensionally Structured Flexible Fog Harvesting Surfaces Inspired by Namib Desert Beetles.

Jun Kyu Park, and Seok Kim

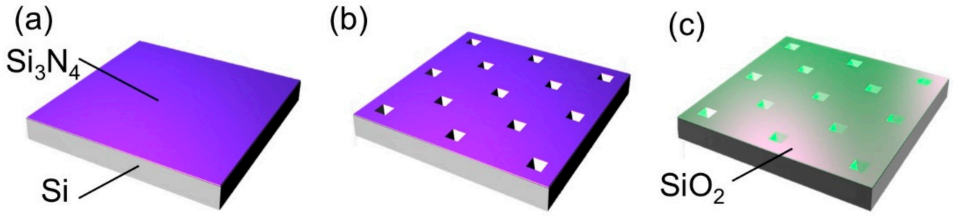

Figure S1. The fabrication procedure to prepare a Si donor substrate with a pyramidal pit array. (**a**) A 1 μm thick low pressure chemical vapor deposition (LPCVD) Si_3N_4 layer on a 500 μm thick Si wafer is patterned by CF_4 plasma etching. (**b**) With the patterned nitride layer as an etch mask, the Si wafer is patterned into a donor substrate with 210 μm deep pyramidal pits by KOH etching. (**c**) The Si_3N_4 layer is removed using hydrofluoric acid and a 300 nm thick SiO_2 layer is thermally grown on the entire Si substrate in a furnace tube at 1100 °C for 6 h. (Figure 2a).

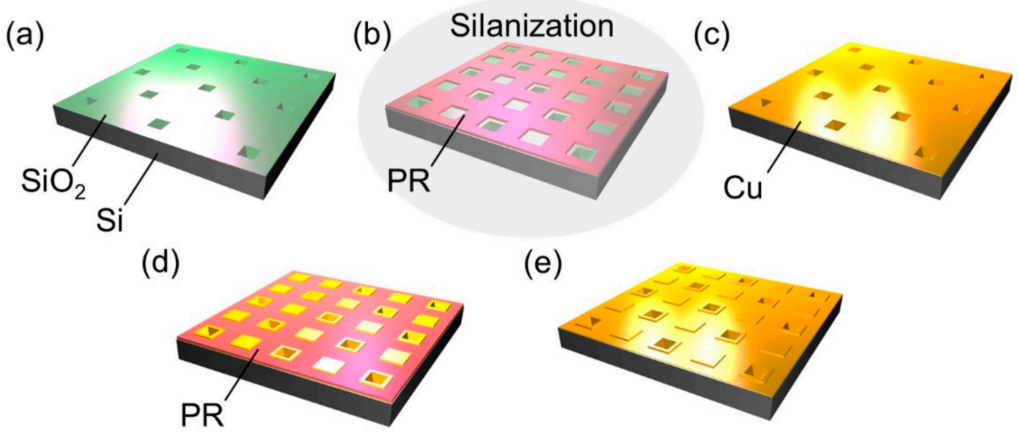

Figure S2. The fabrication procedure to produce a Cu layer with spatially varying thickness. (**a**) The prepared reusable Si donor substrate. (**b**) Trichloro(1H,1H,2H,2H-perfluorooctly)silane is selectively coated on the target areas including pyramidal pits using a patterned AZ5214 photoresist (PR) layer to alleviate the adhesion between Cu and SiO_2. (**c**) After removing the PR, a 100 nm thick Cu seed layer is deposited onto the donor substrate by electron-beam physical vapor deposition. (**d**) Another PR layer is then photolithographically patterned on the Cu seed layer. Using the PR layer, the Cu layer is selectively thickened by electroplating with 30 mA for 1 h. The PR opening is intentionally set to be slightly larger than the pyramidal pit square to prevent cracks from forming on the Cu layer at the edge of pyramidal pits during electroplating. (**e**) The PR is removed, and additional electroplating is performed with 20 mA for 0.5 h.

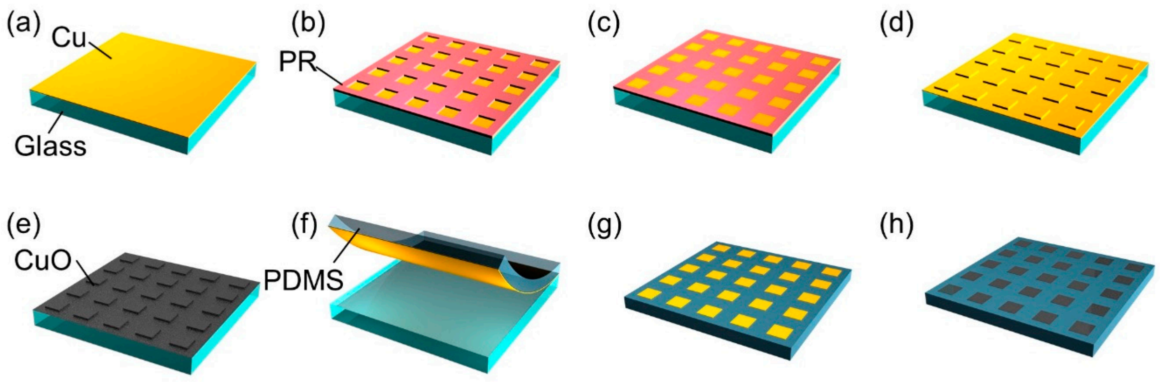

Figure S3. The fabrication procedure to prepare the hybrid surface with a 2D superhydrophilic pattern. (**a**) A 100 nm thick Cu seed layer is deposited onto a glass substrate. (**b**) A PR layer is then photolithographically patterned on the Cu seed layer. (**c**) Using the PR layer, the Cu layer is selectively thickened by electroplating with 30 mA for 1 h. (**d**) The PR is removed, and additional electroplating is performed with 20 mA for 0.5 h. (**e**) Cu is oxidized by alkaline oxidation and CuO-Cu composite layer is formed on the donor substrate. (**f**) A PDMS precursor is poured and cured on the donor substrate to mold a PDMS receiving substrate. The CuO-Cu composite layer is peeled off from the donor substrate and transferred onto the PDMS receiving substrate. (**g**) The Cu pattern is created after timed etching of the CuO-Cu layer on the receiving substrate. (**h**) The additional oxidation converts the Cu pattern to the CuO pattern.

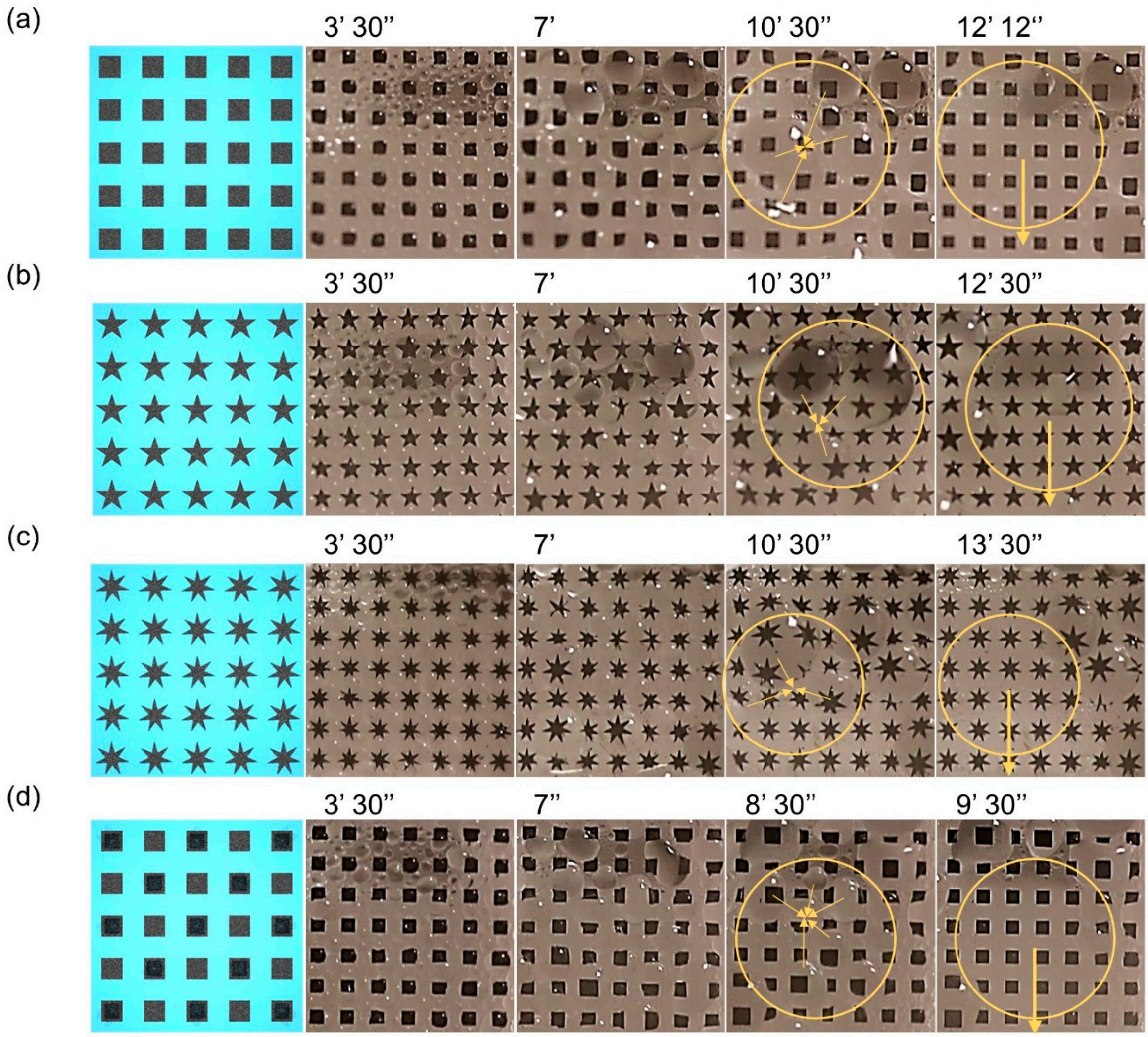

Figure S4. In situ observation of the water collection process of the hybrid surfaces with a sample tilting angle of 90° including square (**a**), 5-star (**b**), 7-star (**c**), and 3D (**d**) pattern samples. Each row has the successive times when images are taken. The last time of each row indicates the departure time when a water droplet starts to fall. Yellow circles present the region where large droplets coalesce (fourth colomn images) and depart (fifth column images).
